# Effects of Ginsenoside Rg1 on the Expression of Toll-Like Receptor 3, 4 and Their Signalling Transduction Factors in the NG108-15 Murine Neuroglial Cell Line

**DOI:** 10.3390/molecules191016925

**Published:** 2014-10-22

**Authors:** Bao-Sheng Zhao, Yang Liu, Xiao-Yan Gao, Hua-Qiang Zhai, Jian-You Guo, Xue-Yong Wang

**Affiliations:** 1 Center of Scientific Experiment, Beijing University of Chinese Medicine, Beijing 100029, China; E-Mails: zhaobs1973@163.com (B.-S.Z.); gaoxiaoyan0913@sina.com (X.-Y.G.); 2 School of Chinese Materia Medica, Beijing University of Chinese Medicine, Beijing 100102, China; E-Mails: netug@126.com (Y.L.); zhaihq@bucm.edu.cn (H.-Q.Z.); 3 Key Laboratory of Mental Health, Institute of Psychology, Chinese Academy of Sciences, Beijing 100101, China; E-Mail: guojy@psych.ac.cn

**Keywords:** Alzheimer’s disease, ginsenoside Rg1, toll-like receptor, nuclear factor kappa B, tumor necrosis factor receptor-associated factor-6

## Abstract

As one of the most important components of *Panax ginseng*, ginsenoside Rg1 has certain anti-aging effects, improving the activity of learning and memory. Studies have showed that ginsenoside Rg1 improves the memory impairment associated with Alzheimer’s disease (AD). In this study, the effects of ginsenoside Rg1 were investigated through the activity of toll-like receptor (TLR) 3, TLR4 and their signaling transduction pathways in amyloid β peptide 25–35 (Aβ_25–35_) induced AD cell model. Thus we investigated several critical components of the TLR pathway. The neuroglial cell line NG108-15 was stimulated with or without Aβ_25–35_, while different concentrations of ginsenoside Rg1 were administered. After 24 h, tumor necrosis factor-α (TNF-α), interferon-β (IFN-β) in cell supernatant and inducible nitric oxide synthase (iNOS) in cell lysate supernatant were measured with enzyme-linked immunosorbent assays (ELISAs). The mRNA and protein expression of TLR3, TLR4, nuclear factor kappa B (NF-κB) and tumor necrosis factor receptor-associated factor-6 (TRAF-6) were detected by real-time PCR and western blot methods, respectively. The experimental results showed that Aβ_25–35_ could markedly raise the level of TNF-α, IFN-β and iNOS, and increase the expressions of mRNA and TLR3, TLR4, NF-κB and TRAF-6 protein in the NG108-15 cells. At the same time, the ginsenoside Rg1 significantly reduced the expressions of proteins and mRNA of TLR3, TLR4, NF-κB and TRAF-6, and down-regulated the levels of TNF-α, IFN-β of cell supernatant and iNOS of cell lysate supernatant in a concentration-dependent manner. In conclusion, ginsenoside Rg1 has good activity for suppressing the signaling transduction pathway of TLR3 and TLR4, and decreasing the inflammation factors induced by Aβ_25–35_ in NG108-15 cells, and this may be the mechanism of ginsenoside Rg1 action in AD treatment, but more studies are needed to identify its specificity.

## 1. Introduction

Alzheimer’s disease (AD), an irreversible and progressive neurodegenerative disease, is characterized with a gradual onset, the advancement of memory impairment, and abnormal cognition and judgment [1]. As is known to all, the prevalence of AD is strongly associated with aging, so with the extension of people’s life expectancy, the number of AD patients has increased accordingly. However, there is still no effective therapy or new drugs to treat AD at present [2]. In the brains of AD patients, the neuropathologic lesions include the deposition of extracellular Aβ in senile plaques, the formation of neurofibrillary tangles, and loss of neurons or synapses, *etc.* [3]. In addition, the activation of the innate immune response by reactive glia is a consistent pathological event in AD [4]. Neuroinflammation in the AD brain is concentrated at the sites of Aβ plaques, which exhibit increased levels of proinflammatory cytokines, proteases and complement components [5]. Activated astrocytes and microglia surround and infiltrate the Aβ plaques, which is believed to be the major source of local inflammatory components [6]. There is an increasing evidence that neuroinflammation plays a major role in AD pathogenesis. For example, in 2001, Weggen *et al.* [7] found that long-term treatment with non-steroidal anti-inflammatory drugs reduced the risk of AD and delayed the progression of AD.

Toll-like receptors (TLRs), are essential signaling components of the mammalian host defense system. At least 12 TLRs were found in rats and 10 TLRs in human beings [8]. In AD patients’ brains, cells express more TLRs than those in the normal brain [9]. For example, TLR 2 and 4 are expressed more in brains of AD patients [10] than in healthy brains. Similarly, TLR1-8 gene expression is obviously increased in the microglia of postmortem tissue from AD patients compared with healthy people [11]. Up-regulating levels of TLR 2 and TLR 7 in murine models of AD also gave the same result [12]. Recent clinical studies showed that TLRs, especially TLR 3 and TLR 4, were significantly increased in AD patients and animal models, and the up-regulated expression of TLR 3 and TLR 4 was of great importance in the pathogenesis and progression of AD [13,14].

Chinese herbs from medicinal plants show certain effects for treating AD [15]. *Panax ginseng* is a well-known Chinese herb used for treating neurodegenerative diseases such as AD [16]. Ginsenoside Rg1, the major component of *Panax ginseng*, can fight against aging, promoting the ability of learning and memory in the normal brain [17]. In addition, it is reported that ginsenoside Rg1 can also improve the memory impairment, and is widely used to treat AD in the clinic [18,19]. However, the mechanism of ginsenoside Rg1 action for treating AD remains unknown. Therefore, in the present study, the effects of ginsenoside Rg1 were examined on an AD Aβ-induced NG108-15 cell line. The TLR3 and TLR4 and their major transduction pathway molecule levels were measured by real-time PCR and western-blot, respectively. The production of inflammatory species such as IFN-β, TNF-α, and iNOS were investigated with enzyme-linked immunoadsorbent assay (ELISA) methods.

## 2. Results and Discussion

### 2.1. Effects of Ginsenoside Rg1 on TNF-α, IFN-β and iNOS Productions

The levels of TNF-α, IFN-β in cell medium and iNOS in cell lysate were markedly increased by Aβ_25–35_ stimulation (all *p* < 0.01). Ginsenoside Rg1 treatment (2 µg/mL) did not affect the levels of TNF-α, IFN-β and iNOS (*p* *>* 0.05). However, 4 to 32 µg/mL of Rg1 treatment significantly decreased the production of TNF-α, IFN-β and iNOS in a concentration-dependent manner, although ginsenoside Rg1 at 4 µg/mL did not affect IFN-β level (Table 1).

**Table 1:** Effects of ginsenoside Rg1 on TNF-α, IFN-β and iNOS production (*x* ± SD, *n* = 8).

Group	Conc. (µg/mL)	TNF-α (pg/mL)	IFN-β (pg/mL)	iNOS (U/L)
Control	—	27.43 ± 8.56	31.57 ± 7.31	1.09 ± 0.18
Aβ_25–35_	—	73.12 ± 15.45 *	92.25 ± 17.82 *	3.67 ± 0.43 *
Rg1/Aβ_25–35_	2	67.48 ± 12.33	86.14 ± 15.33	3.21 ± 0.36
	4	59.21 ± 13.14 ^Δ^	77.09 ± 10.89	2.94 ± 0.28 ^Δ^
	8	51.27 ± 13.06 ^ΔΔ^	72.32 ± 12.46 ^Δ^	2.56 ± 0.31 ^ΔΔ^
	16	46.21 ± 10.58 ^ΔΔ^	61.13 ± 9.33 ^ΔΔ^	2.14 ± 0.29 ^ΔΔ^
	32	42.16 ± 9.31 ^ΔΔ^	52.32 ± 7.56 ^ΔΔ^	1.97 ± 0.18 ^ΔΔ^

Notes: Compared with the vehicle treated control group, * *p* < 0.05, ** *p* < 0.01; Compared with the Aβ_25__–35_ stimulated group, ^Δ^ *p* < 0.05, ^ΔΔ^ *p* < 0.01.

### 2.2. Effects of Ginsenoside Rg1 on the Levels of TLR3, TLR4, NF-κB and TRAF-6 mRNA

**Figure 1:**
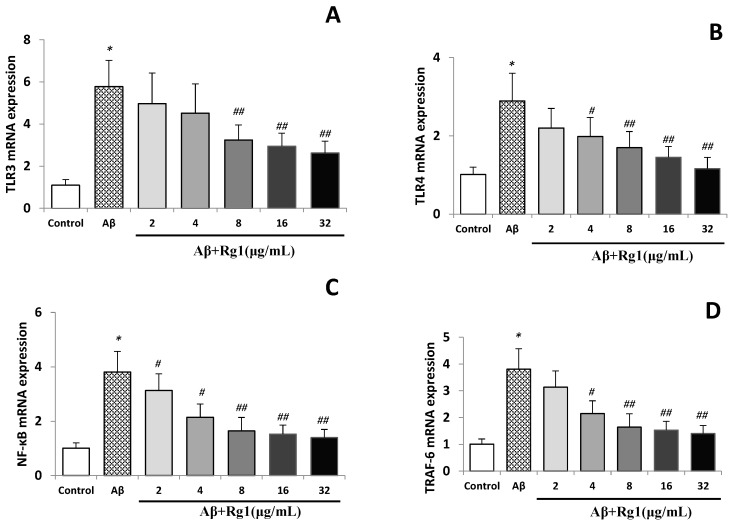
(**A**) Effect of ginsenoside Rg1 on the mRNA levels of TLR3; (**B**) effect of ginsenoside Rg1 on the mRNA levels of TLR4; (**C**) effect of ginsenoside Rg1 on the mRNA Levels of NF-κB; (**D**) effect of ginsenoside Rg1 on the mRNA levels of TRAF-6.

The control group showed weak mRNA TLR3, TLR4, NF-κB and TRAF-6 expression levels, and Aβ_25–35_ significantly elevated the mRNA expressions of TLR3, TLR4, NF-κB and TRAF-6 (all *p* < 0.01). Treatment with ginsenoside Rg1, markedly reduced the TLR3, TLR4, NF-κB and TRAF-6 mRNA expressions induced by Aβ in a concentration-dependent manner (Figure 1A–D). Although 2 µg/mL ginsenoside Rg1 treatment decreased the expression of TLR3, TLR4 and TRAF-6, no statistically significant difference was found (*vs.* Aβ stimulated group). Ginsenoside Rg1 (4 µg/mL) did not affect TLR3 expression (*p* > 0.05), but significantly decreased the expression of TLR4, NF-κB and TRAF-6 (all *p* < 0.05) of Aβ-stimulated cells. Ginsenoside Rg1 at 8, 16 and 32 µg/mL concentrations inhibited the mRNA up-regulated mRNA expression of TLR3, TLR4, NF-κB and TRAF-6 (all *p* < 0.01) (Figure 1) of Aβ-stimulated cells.

Data are expressed as means ± SD. * *p* < 0.01 *vs.* control group,**^ #^** *p* < 0.05, **^##^** *p* < 0.01 *vs.* Aβ-stimulated group.

### 2.3. Effects of Ginsenoside Rg1 on the Protein Expressions of TLR3, TLR4, NF-κB and TRAF-6

**Figure 2:**
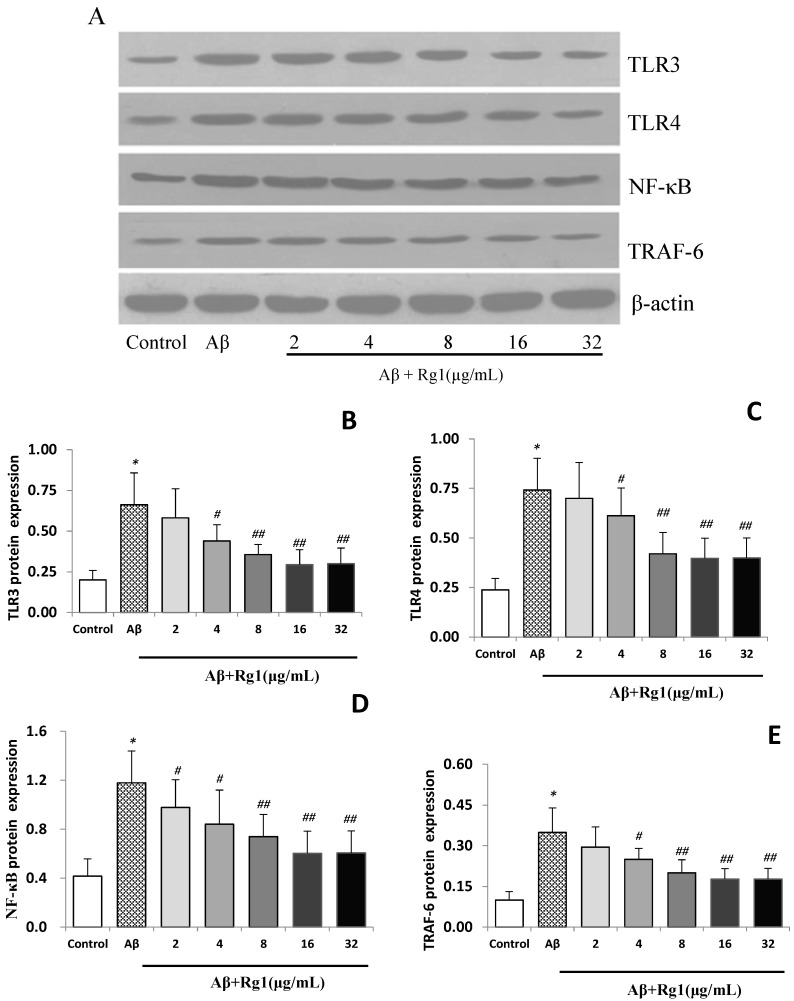
(**A**) Effect of ginsenoside Rg1 on the protein expression of TLR3, TLR4, NF-κB and TRAF-6 (pictures of western-blots); (**B**) effect of ginsenoside Rg1 on the protein expression of TLR3; (**C**) effect of ginsenoside Rg1 on the protein expression of TLR4; (**D**) effect of ginsenoside Rg1 on the protein expression of NF-κB; (**E**) effect of ginsenoside rg1 on the protein expression of TRAF-6.

Similar to the observed gene expression results, weak protein expressions of TLR3, TLR4, NF-κB and TRAF-6 were also detected in the control group (Figure 2A). Aβ_25–35_ stimulation induced significant elevations of protein expressions of TLR3, TLR4, NF-κB and TRAF-6 (all *p* < 0.01). Ginsenoside Rg1 treatment significantly decreased the up-regulated protein expressions of TLR3 and TLR4 which were induced by Aβ in a concentration-dependent manner, although 2 µg/mL ginsenoside Rg1 treatment did not affect the protein levels of TLR3 and TLR4 (*p* > 0.05, Figure 2B,C). However, ginsenoside Rg1 treatment significantly decreased the protein levels of NF-κB and TRAF-6 in Aβ-induced cells at the majority of concentrations tested (except TRAF-6 at 2 µg/mL, Figure 2D,E).

### 2.4. Discussion

The NG108-15 cell line is a hybrid cell line formed from mouse neuroblastoma and rat glioma cell lines [20], and is widely used as a neuron model in electrophysiology and pharmacology research. NG108-15 cell line synthesizes and releases AChE, and also exhibits neurites, ionic channels and enzymes like normal neural cells.

Data are expressed as means ± SD. * *p* < 0.01 *vs.* control group, **^#^** *p* < 0.05, **^##^** *p* < 0.01 *vs.* Aβ-stimulated group.

Therefore, the NG108-15 cell line is a good model system for studying AD [21]. Studies showed that Aβ caused the death of certain primary neuronal cells and other cell lines, and its toxic effects seemed to be dependent on its own ability to aggregate, and that the 25–35 fragment of Aβ appeared to be the proposed active portion [22,23]. Reduced cell survival and increasing AchE activity were observed after Aβ was added to the NG108-15 cells, which was very close to what was seen in AD [24]. Results showed that Aβ_25–35_ markedly increased the levels of TNF-α, IFN-β and iNOS in the cell medium. Ginsenoside Rg1 treatment significantly decreased the content of TNF-α, IFN-β and iNOS which were induced by Aβ in a concentration-dependent manner. In addition, when the NG108-15 cells were stimulated by Aβ, the mRNA and protein expressions of TLR3, TLR4, NF-κB and TRAF-6 were also elevated in the NG108-15 cells. Treatment with ginsenoside Rg1 significantly reduced the protein and mRNA expressions of TLR3, TLR4, NF-κB and TRAF-6 which were induced by Aβ.

Neuroinflammation is found in the AD brain, especially concentrated at sites of Aβ plaques. In the clinic, patients can be administrated non-steroidal anti-inflammatory drugs to reduce the risk of AD, which indicated that neuroinflammation took part in AD pathogenesis [25]. As inflammatory marker production (e.g., TNF-α and IFN-β) increased in Aβ-induced NG108-15 cells, this study confirmed that this cell line could be used to study AD.

TLRs are major transmembrane pattern-recognition receptors (PRRs), which plays a crucial role in initiating immunity against invading microbial pathogens. Each TLR recognizes distinct pathogen-associated molecular patterns (PAMPs) that include lipids, nucleic acids lipoproteins, and proteins. TLRs expressed in a variety of mammalian immune cell types such as NK cells, monocytes, macrophages, dendritic cells, mast cells, neutrophils and endothelial cells [26,27]. TLRs were also presented in the brain, and studies showed that their expression was limited to astrocytes, microglia and oligodendrocytes [28,29]. However, recent studies found that neurons at least express some TLRs [30]. In this study, normal NG108-15 cells expressed TLR3 and TLR4, but the expression is weak compared with that of Aβ-stimulated group. The result showed that certain cells besides immune cells expressed TLRs, and participated in certain neuroimmunology, biology and pathology processes.

All TLRs are located in the cell membrane and have a common expression in the cytosolic domain. Binding specific TLR ligands leads to the recruitment and activation of adapter molecules, such as myeloid differentiation factor 88 (MyD88) which is activated by all TLRs except TLR3 [31]. MyD88-dependent signaling involves TRAF-6 to dissociate the NF-κB inhibitor IκB, which results in translocation of NF-κB into the nucleus, leading to transcription of immunoregulatory genes [32]. TLR3 signaling is MyD88-independent, which results in phosphorylation of IRF-3 and subsequent induction of IFN-β expression. TRIF additionally activates TRAF-6, leading to translocation of NF-κB as described above [33]. Recent studies showed that TLR expression was up-regulated in the AD brain. The TLR3 and TLR4 genes have emerged as candidate susceptibility genes for AD. For example, human brain neurons expressing TLR3 play an important role in initiating an inflammatory reaction in AD [34]. Mutation attenuates TLR4 signals in response to LPS and diminishes the ability to induce inflammation [35]. Ginsenoside Rg1 is one of the most pharmacologically active saponins, even though it is found only in trace amounts. This study focused on identifying the potential mechanisms of ginsenoside Rg1 action on AD. The preliminary study found that a concentration of ginsenoside Rg1 of 32 µg/mL was effective to decrease the TNF-α production in an Aβ-induced AD cell model. However, this concentration of ginsenoside Rg1 also did not affect the cell survival rate in normal NG108-15 cells (data not shown). Therefore, ginsenoside Rg1 concentrations of 2, 4, 8, 16 and 32 µg/mL were chosen in the present study.

We found that normal NG108-15 cells slightly expressed TLR3 and TLR4 mRNA and protein. However, the mRNA and protein levels of TLR3 and TLR4 were significantly elevated when Aβ was added to the cells. Similar changes were also observed in the expressions of NF-κB and TRAF-6, the results of which indicated that TLR3 and TLR4 signaling transduction pathways participated in the inflammation reaction induced by Aβ. The up-regulated expressions of TLR3, TLR4, NF-κB and TRAF-6 were significantly attenuated when the cells were treated with Rg1, indicating that ginsenoside Rg1 might affect TLR3, TLR4 and their signaling transduction pathways.

In conclusion, the present study demonstrated that Aβ_25–35_ up-regulated expressions of TLR3, TLR4, NF-κB and TRAF-6, and elevated the levels of TNF-α, IFN-β and iNOS in NG108-15 cells. This study suggests that the TLR3 and TLR4 transduction pathways participate in the inflammation process in the AD cell model. Ginsenoside Rg1 inhibited the up-regulated expressions of TLR3, TLR4, NF-κB and TRAF-6 both at mRNA and protein levels, and decreased the amount of TNF-α, IFN-β and iNOS. The therapeutical effect of ginsenoside Rg1 on AD might be related to TLR3, TLR4 and their signaling transduction pathways.

In this paper, we emphasized the influence of Aβ on inflammatory factors, TLR3/4 and their signaling transduction pathways. In our preliminary tests, we investigated the influence of Aβ on cell survival rate, but didn’t examine the influence on cell viability. Whether ginsenoside Rg1 influences the viability of cells and other TLRs signaling pathways or not remains unclear and needs further investigation.

## 3. Experimental Section

### 3.1. Chemicals and Reagents

Ginsenoside Rg1 was purchased from the National Institute for the Control of Pharmaceutical and Biological Products (Beijing, China), DMEM media and fetal bovine serum (FBS) were purchased from Gibco (Carlsbad, CA, USA). Primers of TLR3, TLR4, NF-κB, TRAF-6 and β-actin were synthesized by Invitrigen Life Technology Co. Ltd. (Shanghai, China), and M-MLV Reverse Transcriptase was obtained from Promega (Beijing, China) Biotechnology Co. Ltd. (Beijing, China), and primary and second antibodies of TLR3, TLR4, NF-κB, TRAF-6, β-actin were produced by Santa Cruz (Santa Cruz, CA, USA). TNF-α, INF-β and iNOS ELISA kits were purchased from R&D Co., Ltd. (Shanghai, China). All other chemicals were obtained from Sigma-Aldrich Chemical Co. (St. Louis, MO, USA), unless otherwise stated.

### 3.2. Cell Culture and Treatments

Neuroglial cell line NG108-15 obtained from the Institute of Basic Medical Sciences, Chinese Academy of Medical Sciences (Beijing, China) was cultured in DMEM media supplemented with 10% (v/v) heat inactivated fetal calf serum, penicillin (100 U/L), streptomycin (100 mg/L) and NaHCO_3_ (2 g/L). Cells were maintained in a humidified 37 °C incubator with a 5% CO_2_ and 95% air and used for assays during the exponential growth phase.

Cells were stimulated with or without Aβ_25–35_ (5 µmol/mL, the best stimulating concentration which was pretested in a previous study, data not shown) at 2 × 10^5^ cells/mL, and ginsenoside Rg1 was added to the cells at final concentrations of 2, 4, 8, 16 and 32 µg/mL, respectively. In the control group, cells were treated with vehicle alone. After incubation for 24 h, the cell supernatant was collected, and levels of TNF-α, IFN-β were measured with ELISA kits. Then the cells were lysed and centrifuged, and the supernatant of cell lysate was obtained and iNOS was measured with the ELISA method. In another experiment, the cells were harvested, and the mRNA and protein levels of TLR3 and TLR4 were analyzed by real-time PCR and western blot, respectively. The concentration of Aβ_25–35_ in 5 µmol/L has been reported to reduce cell survival and increase acetylcholinesterase (AchE) activity in NG108-15 cells [21].

TNF-α, IFN-β and iNOS Assay After 24 h incubation, TNF-α and IFN-β in the medium and iNOS in cells were measured by ELISA with an R&D System ELISA kit as described previously [36]. In brief, 96-well plates containing anti-TNF-α, anti-IFN-β antibody and anti-iNOS antibody were incubated with supernatant samples, or cell lysate (100 µL/well), or TNF-α, IFN-β and iNOS standards for 1 h at 37 °C. The supernatant was discarded and the plates were washed five times with washing solution. After having been incubated for 30 min at 37 °C with streptavidin-HRP, the plates were washed five times again and incubated with TMB substrate for 10 min at 37 °C. At last, stop solution was added into the plates (100 µL/well), then the absorbance was measured at 450 nm with an enzyme immunoassay instrument (Model 550, Bio-Rad, Hercules, CA, USA).

### 3.3. Real-Time PCR Measurement

The total RNA was extracted from NG108-15 cells using the TRIzol method as described previously [37]. Briefly, the quantity and quality of the different samples of RNA were determined by the 260/280 nm absorbance ratio, then RNA reversely transcribed to cDNA using the Taqman Reverse Transcription Reagents. Reverse transcription was performed at 42 °C for 30 min followed by RT inactivation at 94 °C for 5 min. All samples were operated in triplicate, and the relative expression values were normalized to the expression value of β-actin (β-actin as the calibrator gene. The sequences for sense (F) and anti-sense (R) primers were shown in Table 2.

**Table 2:** The primer sequences of TLRs and their components of downstream signaling transduction pathway.

	Sense (F)	Anti-Sense (R)
**TLR3**	tGCCTTGGTCCCAAGCCTTCAACGA	TGGCCCGAAAACCTTCTTCTCAACGGA
**TLR4**	CGCTTTCAGCTTTGCCTTCATTAC	TGCTACTTCCTTGTGCCCTGTGAG
**NF-κB**	GCTCGGCTGAATGAATCTACC	GTCTCCACGTATTTCCGCAACT
**TRAF-6**	CAGGGATATGATGTGGAGT	TACCCTCAGGGAAAGAAT
**β-actin**	GTACCCCAGCATTGCTGACA	CTCCTGCTTGCTCATCCACATC

All primers were synthesized by Invitrogen (Carlsbad, CA, USA). Real-time PCR assay was performed on a ABI 7300 Real-time PCR meter (ABI, New York, NY, USA). All reactions were performed in a total of 20 µL including 1× IQ SYBR Green Supermix from Takara (Dalian, China), 250 nM of each primer and 2 µL cDNA as template. PCR conditions were as follows: 95 °C for 30 s to activate the iTaq polymerase, 45 cycles of 5 s at 95 °C and 31 s at 60 °C. Melt curve was performed for all samples in which only sharp melting points were observed, indicating a specific signal and no primer dimers or mispriming. Relative contents of the TLR3, TLR4, NF-κB and TRAF-6 genes were calculated using the ddCt method (2^−ddCt^) as described by Schmittgen *et al.* [38].

### 3.4. Western Blotting Analysis

Cells were lysed by adding 200–500 µL ice-cold lysis buffer (20 mM HEPES, pH 7.4, 150 mM NaCl, 1% Triton X-100, 0.2 mg/mL benzamidine, 0.1 mg/mL leupeptin and 0.5 mM phenylmethyl sulphonylfuoride) to cells monolayers. Insoluble cell fractions were then pelleted by centrifugation in a microcentrifuge at 20,000 rpm and 4 °C for 5 min, and the supernatant including the cytosolic extract was run onto 10% SDS-PAGE, then the proteins were transferred to a nitrocellulose membrane (Millipore, Bedford, MA, USA) using Towbin transfer Buffer. After incubating membranes in blocking buffer (5% low-fat milk in 100 mM Tris-HCl (pH 7.5), NaCl (145 mM), and 0.05% Tween 20; TBST), primary antibodies were added, and membranes were incubated overnight at 4 °C with gentle rocking. Membranes were washed with the buffer solution and incubated with secondary horseradish peroxidase-conjugated antibody for 1 h at room temperature. Immunoreactivity was visualized by ECL. Protein expressions of TLR3, TLR4, NF-κB and TRAF-6 were quantified by densitometry using the Scion Image Beta 4.02 software and are shown as density relative to β-actin as described by Lou *et al.* [39].

### 3.5. Statistical Analysis

All data were expressed as *x* ± SD. The statistical significance between groups was calculated by one-way ANOVA followed by the Bonferroni *post hoc* tests for multiple comparisons. Differences were considered statistically significant at *p* < 0.05.

## 4. Conclusions

In conclusion, the results demonstrated that ginsenoside Rg1 has a certain anti-AD activity whose possible mechanism may be related to suppressing the signaling transduction pathway of TLR3 and TLR4, and decreasing the inflammation factors induced by Aβ_25–35_ in NG108-15 cells.
